# Infectious Diseases Fellowship Training in Caring for People Who Use Drugs: A National Assessment of an Emerging Training Need

**DOI:** 10.1093/ofid/ofae544

**Published:** 2024-09-24

**Authors:** Shilpa Vasishta, Raagini Jawa, Sarah Kurz, Nathanial S Nolan

**Affiliations:** Division of Infectious Diseases, Montefiore Medical Center, New York, New York, USA; Center for Research on Healthcare, Division of General Internal Medicine, School of Medicine, University of Pittsburgh, Pittsburgh, Pennsylvania, USA; Division of Infectious Diseases, Department of Internal Medicine, Michigan Medicine, Ann Arbor, Michigan, USA; Division of Infectious Disease, Veterans Affairs St Louis Health Care, St Louis, Missouri, USA; Division of Infectious Diseases, Washington University School of Medicine, St Louis, Missouri, USA

**Keywords:** addiction, fellowship, risk reduction, substance use

## Abstract

**Background:**

Infectious complications of substance use are increasingly encountered in infectious diseases (ID) clinical care. In this study, we surveyed ID fellows in the United States to assess training experiences, clinical practices, and perspectives in caring for people who use drugs (PWUD).

**Methods:**

An 18-item survey was distributed to ID fellows via email and social media platforms. Four focus groups were conducted with a subset of participants to elucidate themes in fellowship experiences and training needs.

**Results:**

Among 196 survey respondents (estimated 24% response rate), all reported caring for PWUD in ID fellowship. Forty-nine percent received some formal fellowship-based didactics around care of PWUD, and 64% worked with faculty seen as advocates for PWUD. Integrated care practices for PWUD were infrequently employed: 50% offered risk reduction counseling, and 33% discussed medications for opioid use disorders, naloxone, or HIV preexposure prophylaxis. Risk reduction counseling was felt to be “extremely” within scope of ID practice (69%), although comfort level with this skill was low; fellows’ likelihood of offering counseling was significantly associated with their comfort (*P* ≤ .0001). Common themes in caring for PWUD included an expanded role for ID consultants, a lack of formalized training, and benefits of developing dedicated opportunities in this field.

**Conclusions:**

ID fellows frequently care for PWUD but may have gaps in knowledge or comfort that affect care practices. Increased fellowship training in the care of PWUD has potential to improve clinical practices and support growing interest in this field among current and prospective ID fellows.

## Teaser

US infectious diseases fellows often care for people who use drugs, but they report limited experience in integrated infectious diseases/addiction care as well as a lack of comfort in these skills. Increased fellowship-based training would be beneficial in meeting fellows’ interests and improving clinical care.

Infectious complications of substance use represent a growing component of infectious diseases (ID) clinical practice [1–5]. Since 2010, the US opioid epidemic has been associated with a 4-fold rise in incident hepatitis C [1], clustered outbreaks of HIV [2, 3], and a nearly 10-fold increase in serious bacterial infections such as infective endocarditis [4, 5]. Though most US ID clinicians report routinely caring for people who use drugs (PWUD), many practice in settings without dedicated addiction medicine services [6].

Professional societies, including the Infectious Diseases Society of America and HIV Medicine Association, have urged increased training in substance use disorder (SUD) treatment among ID specialists and proposed that aspects of integrated SUD care, such as risk reduction counseling, be considered core competencies in general ID practice [7]. However, <50% of surveyed ID clinicians report counseling their patients on safer use practices [8], and far fewer prescribe medications for opioid use disorder (MOUD) [8, 9]. These findings suggest that individuals presenting with infectious complications of substance use may experience unmet medical needs or missed opportunities for future risk reduction.

Given the rising rates of substance use–related infections [1–5], current ID fellows are likely to manage a greater volume of infections among PWUD as compared with prior groups of graduating ID clinicians. This represents an emerging training need that has not been previously characterized among US training programs. Current fellows, who have trained during the opioid epidemic, also bring a unique generational perspective on the impacts of substance use on individuals and communities, which could be informative for training programs and the field of ID. We surveyed and interviewed a geographically diverse sample of ID fellows to assess training experiences, clinical practices, and perspectives in the care of PWUD.

## METHODS

We developed a survey to characterize US ID fellows’ experiences in caring for PWUD. Items were developed by the authors and then iteratively reviewed by 7 ID and addiction medicine specialists to refine questions and survey flow. Cognitive interviews were conducted with 7 current and recent ID fellows to ensure relevance and comprehensibility.

The finalized anonymous survey tool included 18 multiple-choice, open-ended, or matrix-style questions (Supplementary 1). Survey questions elicited information regarding participant demographics, fellowship program characteristics (including the presence of formal didactics in the care of PWUD or a faculty advocate for PWUD, as perceived by fellows), the frequency of carrying out select clinical practices (eg, risk reduction counseling, discussion of MOUD or HIV preexposure prophylaxis), attitudes toward select clinical practices (ie, comfort level with each practice, degree to which each practice was perceived to be within the scope of ID), and local resources. The survey was deemed exempt by the Washington University institutional review board (202212115).

ID fellows in US training programs as of January 2023 were eligible to participate. The total eligible population was estimated at 820 based on National Resident Match Program data [10] from 2021 (365 matched fellows) and 2022 (358 matched fellows) and individual program rosters of advanced-year fellows (97 listed among 117 program websites with available data).

Survey recruitment was conducted through emails to fellowship program directors for internal dissemination, based on publicly available contact information (n = 163). To maximize participation, recruitment was also conducted through social media platforms (X [formerly Twitter] and Mastodon) and the Infectious Diseases Society of America message board; participants were provided study inclusion criteria upon accessing the survey and invited to proceed if self-identifying as eligible. The survey was administered via Qualtrics. Data collection occurred for 3 weeks in January 2023. There was no incentive for survey participation.

Categorical variables were analyzed descriptively. We then evaluated associations among fellow or program features (eg, year of training, presence of fellowship-based didactics), the likelihood of carrying out clinical practices of interest, and attitudes toward clinical practices. These analyses were conducted via chi-square test with correction for multiplicity. Analyses were conducted with SAS version 9.4 (SAS Institute).

To elucidate themes that were less readily captured via survey responses, qualitative data were collected through focus groups. All fellows who completed the survey were invited to participate in a focus group, and all fellows who accepted the invitation were scheduled in 1 of 4 groups based on availability. Focus groups were conducted in 60-minute virtual sessions over Zoom (Zoom Video Communications, Inc) [11] via a standardized interview guide developed by the authors based on questions and themes suggested by the survey responses (Supplementary 2). Participants gave consent at the start of each session and were offered inclusion in a $25 gift card raffle as an incentive. Focus groups were recorded and transcribed in a deidentified manner with Otter (Otter.ai). Transcripts were reviewed after each session by 2 authors (S. V., N. S. N.), and a codebook was derived inductively to highlight themes in responses (Supplementary 3). Further recruitment beyond the initial 4 sessions was deferred due to thematic saturation. Transcripts were coded independently by 2 authors (S. V., N. S. N.) with Dedoose version 9.0.107 (Dedoose) and jointly reviewed to adjudicate discrepancies. Primary themes and illustrative quotes were extracted.

## RESULTS

### Survey

#### Participant and Program Characteristics

An overall 196 fellows completed the survey (estimated 24% response rate). Participants were primarily first-year (n = 95, 49%) and second-year (n = 83, 42%) ID fellows in university-based training programs (n = 170, 87%) of broad geographic spread. Data regarding gender identity, race, and ethnicity are noted in Table 1. All participants reported caring for PWUD in fellowship, with many reporting a significant clinical volume of >10 patients per month (n = 86, 44%). Most indicated receiving informal teaching in the care of PWUD during clinical encounters (n = 148, 81%), while half cited formal fellowship-based didactics (n = 96, 49%). Approximately two-thirds identified an ID faculty member in their program who was perceived as an advocate for PWUD (n = 126, 64%).

**Table 1.** Participant Demographics and ID Fellowship Program Characteristics (N = 196)

	No. (%)
ID fellowship	
Year 1	95 (48)
Year 2	83 (42)
Year ≥3	18 (9)
Gender identity	
Female	115 (59)
Male	81 (41)
Race	
White	112 (57)
Asian	52 (27)
Black or African American	3 (2)
Other or multiple	25 (13)
Prefer not to say	4 (2)
Ethnicity	
Hispanic	24 (12)
Not Hispanic	170 (87)
Prefer not to say	2 (1)
Geographic region [12]	
Northeast	62 (32)
Midwest	58 (30)
South	48 (24)
West	28 (14)
Training program type [13]	
University based	170 (87)
Community based, university affiliated	17 (9)
Community based	6 (3)
Other	1 (1)
Monthly practice volume of PWUD	
None that I know of	0
1–5 patients	56 (29)
6–10 patients	52 (27)
>10 patients	86 (44)
Prefellowship SUD training	
Medical school-based training	87 (44)
Residency-based training	160 (82)
Buprenorphine waiver training	32 (16)
Other	4 (2)
None	16 (8)
Fellowship SUD training	
Informal teaching during patient care	158 (81)
Fellowship-based lectures or didactics	96 (49)
Independent reading or coursework	89 (45)
Experience treating chronic hepatitis C	92 (47)
Other	3 (2)
None	10 (5)
ID faculty advocate for PWUD within fellowship program: yes	126 (64)

Abbreviations: ID, infectious diseases; PWUD, people who use drugs; SUD, substance use disorder.

#### Clinical Practices

**Figure 1.**
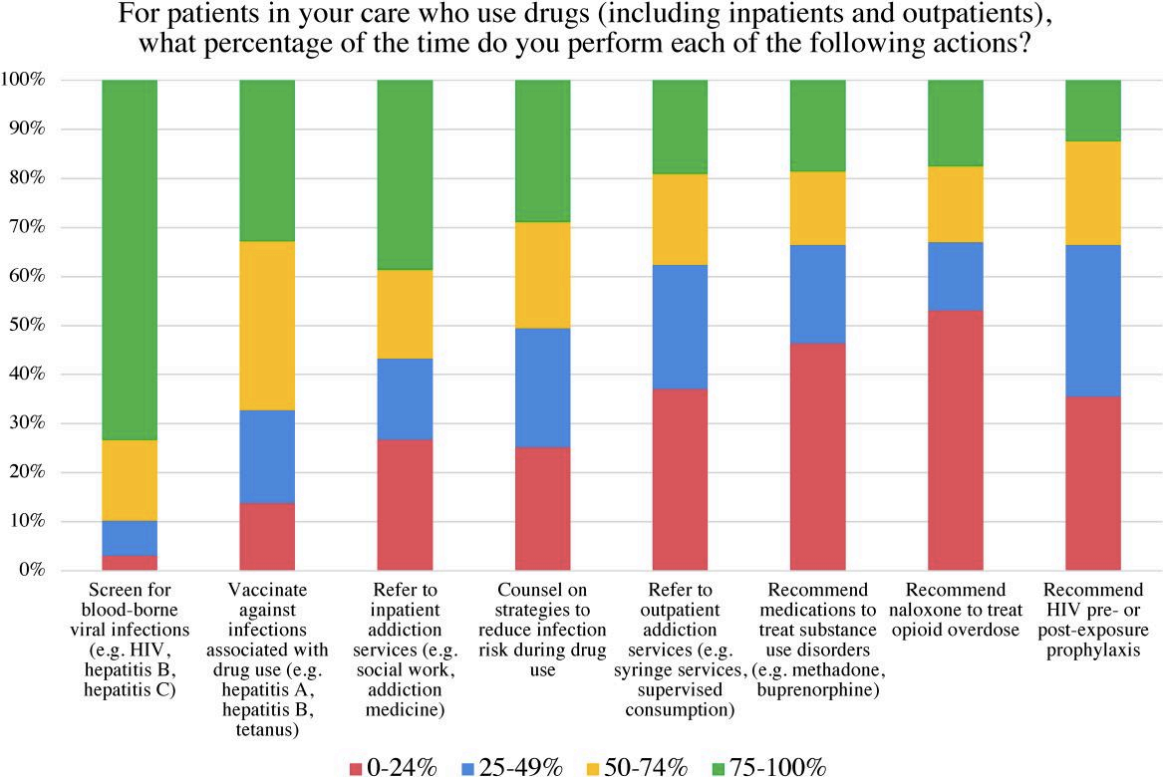
Frequency of clinical practices.

Most participants reported typically (during ≥50% of clinical encounters) screening for bloodborne viral infections (n = 175, 89%), providing vaccinations (n = 131, 67%), and referring to inpatient addiction services (n = 110, 56%) when caring for PWUD. Fewer indicated counseling on strategies to reduce infection risk (n = 98, 50%) or referring to outpatient addiction services (n = 73, 37%). Approximately one-third recommended MOUD (n = 65, 33%), naloxone (n = 64, 33%), or HIV preexposure prophylaxis (n = 65, 33%; Figure 1).

The likelihood of carrying out these clinical practices did not vary significantly by year of training or monthly clinical volume. Participants who reported receiving formal didactics in the care of PWUD were more likely to report referring patients to inpatient addiction services (*P* = .0016). Participants who indicated working with a faculty advocate for PWUD were more likely to report risk reduction counseling (*P* = .0014), recommending MOUD (*P* = .0011) or naloxone (*P* = .0001), and referring to outpatient addiction resources (*P* = .0013).

#### Attitudes: Comfort and Scope

**Figure 2.**
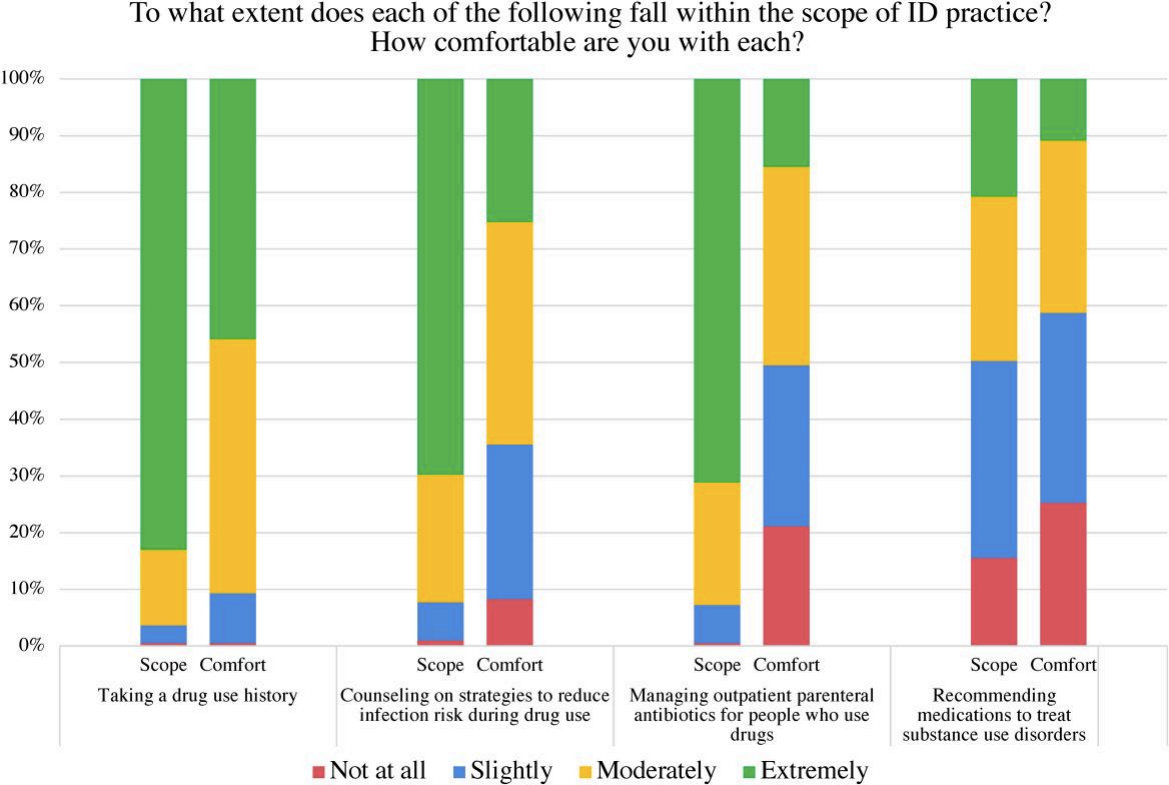
Perceptions of comfort and scope. ID, infectious diseases.

When asked about attitudes toward specific clinical practices, most respondents categorized taking a drug use history (n = 161, 82%) and counseling on risk reduction strategies (n = 136, 69%) as extremely within the scope of ID practice; similar cumulative proportions felt either moderately (n = 176, 89%) or extremely (n = 125, 64%) comfortable with these 2 skills. For outpatient parenteral antibiotic management in PWUD, 138 (70%) rated this skill as extremely within their scope, while fewer felt moderately or extremely comfortable (n = 98, 50%) and some (21%) felt not at all comfortable. A minority of respondents thought that recommending MOUD was extremely within the scope of ID practice (n = 40, 20%); a similar proportion (n = 49, 25%) felt not at all comfortable with this skill (Figure 2).

Neither comfort level nor perception of scope for any of the queried skills varied significantly by year of training or monthly clinical volume of PWUD. Fellows’ self-reported likelihood of offering risk reduction counseling to PWUD was significantly associated with their comfort levels with that skill (*P* ≤ .0001) and not with their perceptions of this skill falling within the scope of ID. Self-reported likelihood of recommending MOUD for PWUD was significantly associated with comfort level (*P* ≤ .0001) and perception of scope (*P* < .0001).

### Resources

**Figure 3.**
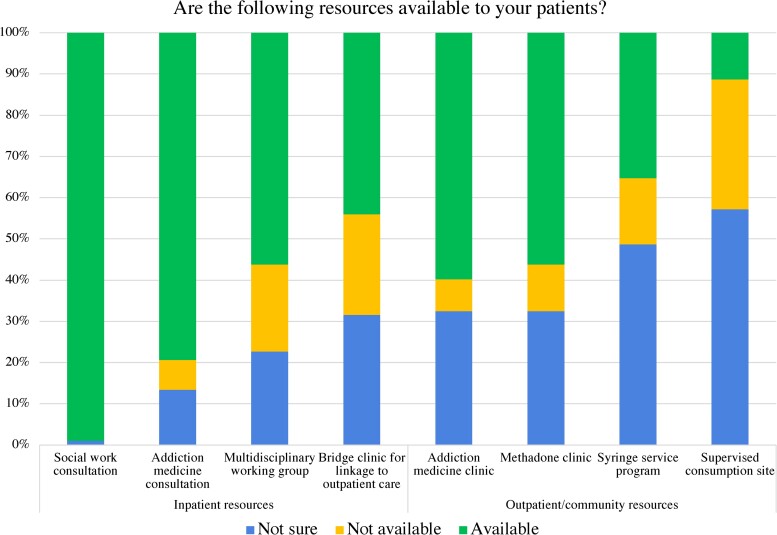
Institutional and community resources.

Most respondents reported institutional availability of inpatient social work consultation (n = 192, 98%) and inpatient addiction medicine consultation (n = 154, 79%). Approximately half noted the availability of a multidisciplinary working group (eg, an endocarditis team or discharge planning team for PWUD; n = 109, 56%), bridge clinic (n = 85, 43%), addiction clinic (n = 116, 59%), integrated primary care clinic (n = 103, 54%), or methadone clinic (n = 109, 56%). Few respondents cited availability of a syringe service program (n = 68, 35%) or supervised consumption site (n = 22, 11%); many were unsure regarding the presence of either resource (n = 94 [48%] for syringe services and n = 111 [57%] for supervised consumption sites; Figure 3).

### Focus Groups

Four focus groups were conducted from March 2023 to September 2023; there were 1 to 3 participants per group and 9 participants in total. Participants primarily identified as second-year fellows (5/9, 55%) of female gender (8/9, 88%) from academic training programs (8/9, 88%) and represented 4 of 4 geographic regions. Three major themes were identified and are discussed in turn.

#### Theme 1: Expanded ID Role

Fellows described an expanded role when caring for PWUD. This included increased attention to preventive health, such as ID screening and vaccination, and an increased focus on advocacy, such as ensuring a multidisciplinary approach to care that included treatment of SUD. One fellow noted, “We tend to be the biggest advocate for these patients. So I think . . . if I’m approaching a patient who uses drugs, I am taking each case . . . a little more personally and feel like I need to be their voice with the other providers.” Fellows described a need to approach management of infections holistically and, at times, modify standards of care to meet the unique needs of PWUDs. One participant stated, “I first try to offer the standard of care . . . the same treatment that I would offer to anybody else . . . [but] then get creative with solutions that might help with more of a patient-centered approach to either improve adherence or meet the patient where they’re at.”

#### Theme 2: Educational Needs

Fellows generally reported a lack of formal training in the care of PWUD in residency and fellowship, with most learning informally through patient care experiences and independent reading. Some discussed feeling incompletely equipped to provide optimal care to their patients, with 1 participant stating, “That's one of the problems with training . . . you might not know the resources that are even available, and you’re the doctor taking care of the patient!” Fellows described variable experiences in learning from faculty about caring for PWUD, with some faculty coming “from a place of allyship” and others in “more [of] a paternalistic role.” Fellows discussed the benefits of building curriculum around the care of PWUD to further clinical care across multiple domains (Table 2), as well as dedicated fellowship tracks in ID and addiction medicine to nurture existing interest and potentially draw more applicants to ID. One fellow elaborated, “Within ID, especially in the state of the match and everything right now, if there were more opportunities, then maybe there would be some more interest, and people kind of making their way to ID fellowship with this sort of focus.”

**Table 2.** Domains of Clinical Practice to Be Supported With Fellowship-Based Training

Comfort in discussing drug use	“It’s almost like asking a sexual history, where I think, until you start doing it more, you feel awkward . . . like am I asking the right questions?”
Knowledge regarding drug use	“That was something that I had to learn about . . . like, the process of injecting yourself with heroin . . . to be able to be more knowledgeable in asking those questions.”
Basic addiction management	“[Suboxone] would be one thing, for example, that I wish I had in my toolkit.”
Resources for people who use drugs	“I had to learn on the fly, what kind of resources are available, where the safe injection sites are, where you can get syringe exchange.”
Coping with difficult cases	“I think it is really important, early on in fellowship, to have that conversation and a safe space . . . because some of those conversations can be really heated or challenging or emotional.”

#### Theme 3: Impacts on Morale

All fellows described caring for PWUD to have positive and negative impacts on their morale. Some endorsed frustration with the limitations faced by their patients and the extra effort required to coordinate care. One in particular said that caring for PWUD takes “a tremendous amount of work, it often feels underappreciated, and sometimes, despite all best efforts, it really doesn’t work . . . and there have definitely been moments where I feel angry.” Other fellows described feeling a strong sense of purpose in caring for PWUD, highlighting the unexpected ID specialist's role in engaging PWUD during their encounters. One participant elaborated that “it's always a really big opportunity, a moment in people's lives and I’ve just been really humbled to be part of that experience.” Fellows also described feeling rewarded to be part of a specialty that shows leadership in improving care for PWUD, with 1 fellow stating, “I think it highlights how important our field is.”

## DISCUSSION

We have conducted a nationwide assessment of ID fellowship training in the care of PWUD, capturing an estimated one-fourth of fellows in training at the time of our study. We find that fellows frequently care for PWUD but most receive limited formalized training in integrated care of ID and substance use. This has potential implications for fellows’ future clinical practices, as well as their sense of efficacy, preparedness, and morale. Given the increasing volume of infections in PWUD seen in ID practice, a lack of comfort and proficiency as reported by many ID fellows may represent an opportunity for improvement among training programs.

An important finding in our survey is the low national uptake of preventive care practices for PWUD, with fewer than half of fellows engaging in preexposure prophylaxis prescription, risk reduction counseling, community resource referrals, or discussions of MOUD. As fellows practice under supervision, these findings provide insight on not only individual trainee practices but also faculty and institutional practices. Among fellows, risk reduction counseling in particular did not appear to be limited by interest or ownership, as this skill was perceived as highly within the scope of ID practice; rather, fellows primarily reported a lack of comfort. Encouragingly, our survey highlights potential facilitators in engaging fellows in these skills. Fellowship-based didactics were associated with more frequent referrals to addiction services, and the presence of a faculty advocate was associated with more frequent risk reduction counseling and discussion of MOUD. Given these results, we believe that a mix of formal education with the opportunity to work with faculty champions who model care at the bedside has the potential to promote durable practice change.

In our focus groups, the lack of formalized training in care of PWUD was a consistent theme. However, caring for PWUD was noted to be a uniquely rewarding facet of ID fellowship, and many felt that increased training opportunities in the intersection of ID and SUD represented a positive direction for the field. Prior surveys of trainees have shown that many are drawn to ID for its social mission and advocacy [14]. As previously noted, aligning ID teaching in response to societal needs has the potential to raise interest in the field and increase a sense of meaning for those practicing within it [15]. Fellows in our survey had varying perspectives on specific competencies, such as prescribing MOUD. Even so, given a strong association between appropriate MOUD prescribing and improved outcomes for conditions such bacterial infections, hepatitis C, and HIV [7], ID fellowship could be a high-impact setting in which to make such training available to those fellows who seek to incorporate MOUD prescribing into their future practice. While some institutions have very well-established addiction medicine rotations and joint ID/addiction tracks [16, 17], our focus groups highlight the importance of building on these opportunities for interested fellows nationwide.

Our study has several limitations. Our survey was voluntary and had potential for selection bias, whereby fellows with specific interests in care of PWUD or those enrolled in programs caring for higher volumes of PWUD may have been more likely to participate. This is important in contextualizing findings, such as participation in fellowship-based didactics among nearly 50% surveyed, which may not be nationally representative. Although we sought to capture perspectives from a diverse group of fellows, certain groups remained less represented in our cohort, including fellows in community-based training programs and fellows from racial groups underrepresented in medicine (Black, Pacific Island/Alaska Native). The self-report design of our study also introduces important limitations, such as social desirability or availability bias, whereby fellows may have overreported specific practices or interests based on perceived goals of the survey or the salience of content queried. This is particularly notable in the focus group portion, where live discussion among a small cohort of fellows, with the presence and facilitation of study investigators, may have inadvertently encouraged bias toward a consensus and early thematic saturation. Despite the potential reporting biases and possible overrepresentation of trainees with addiction medicine interests in larger academic centers, significant gaps in practice were still reported.

Several important issues could not be addressed within the scope of this study but will be important in contextualizing and operationalizing the findings. Although clinical practice was considered in aggregate for this study, ID clinicians occupy diverse clinical roles (eg, inpatient consultation vs HIV primary care) and practice in settings with varying substance use epidemiology, which may warrant differing approaches to SUD care. Moreover, as the ID field navigates challenges with recruitment and workforce, the impact of time constraints, clinical volume, and lack of compensation for integrated care roles remains a critical area for discussion and reform. For ID fellowship programs, implementing novel curricula comes with significant resource needs, including faculty development, institutional collaboration, and curricular time. Given prior studies showing strong variability in practices based on length of time in practice and geographic location [6, 9], there may be benefit to consolidating up-to-date national expertise into formalized curricular resources, as have been developed in other content areas, such as HIV and antibiotic stewardship [18, 19]. Finally, while this survey focused on perspectives of current ID fellows, we recognize that many other stakeholder voices—such as those of program directors, postgraduate ID clinicians, interdisciplinary team members, and patients—are needed to develop a robust vision for optimal training in this realm.

## CONCLUSION

ID fellows frequently care for PWUD but infrequently incorporate integrated care practices, such as risk reduction counseling, in part due to a lack of knowledge and comfort. There is strong interest from fellows in receiving formalized training in the care of PWUD, and there are potential benefits of such training for patient care, fellow morale, and recruitment to the field. ID fellowships may benefit from harnessing local resources to develop dedicated training opportunities and by consolidating national experience and expertise into curricular resources.

## Supplementary Material

ofae544_Supplementary_Data

## Supplementary Data

Supplementary materials are available at *Open Forum Infectious Diseases* online. Consisting of data provided by the authors to benefit the reader, the posted materials are not copyedited and are the sole responsibility of the authors, so questions or comments should be addressed to the corresponding author.

## Notes

***Acknowledgments.*** We acknowledge the following individuals for their contributions to this article: for expert content review, Drs Laura Bamford, Michael Durkin, Leah Harvey, Karim Khan, Simeon Kimmel, Laura Marks, and Jessica Meissner; for survey creation and distribution guidance, Drs Varun Phadke and Judy Shea; for survey piloting and feedback, Drs Tyler Degener, Srinivasa Gopalasamy, Audrey Li, Sophie Rosenmoss, Jonathan Ryder, Christopher Vo, and Carlie Wilson; for statistical support, Dr Emilia Bagiella.

***Author contribution.*** All authors jointly contributed to the design and implementation of the study, analysis of results, and writing of the manuscript.

***Financial support*.** This work was supported in part by the National Institute on Drug Abuse (K12DA050607-03 to R. J.).
