# Supplementary material for: Infectious Diseases Fellowship Training in Caring for People Who Use Drugs: A National Assessment of an Emerging Training Need
Source: Open Forum Infect Dis. 2024 Sep 24;11(10):ofae544. doi: 10.1093/ofid/ofae544 (PMC11474980; doi:10.1093/ofid/ofae544)
Supplement: ofae544_Supplementary_Data [file ofae544_supplementary_data.zip › Supplement 1. Survey form.docx]

Supplement 1. Survey form

The following survey is intended for current Infectious Diseases (ID) fellows to gather insight into practice and training experiences in caring for people who use drugs. Participation is anonymous and voluntary.

For this survey, we ask respondents to consider drug use as broadly encompassing uptake of non-prescribed substances through routes including injection (intravenous, subcutaneous, intramuscular), mucosal administration (nasal, sublingual, rectal), and/or inhalation.

—

Demographics:

What is your current year of ID fellowship? (select one)

- Year 1
- Year 2
- Year 3 or more

In which geographic region is your fellowship? (select one)

- New England (CT, MA, ME, NH, RI, VT)
- Mid-Atlantic (NJ, NY, PA)
- South-Atlantic (DC, DE, FL, GA, MD, NC, SC, VA, WV)
- East South Central (AL, KY, MS, TN)
- West South Central (AR, LA, OK, TX)
- East North Central (IL, IN, MI, OH, WI)
- West North Central (IO, KS, MO, MN, ND, NE, SD)
- Mountain (AZ, CO, ID, MT, NM, NV, UT, WY)
- Pacific (AK, CA, HI, OR, WA)

What is your fellowship program type? (select one)

- University-based
- Community-based
- Community-based/university-affiliated
- Military-based
- Other

Are you part of a training “track” in your fellowship? If so, please indicate the type/focus (select all that apply)

- Transplant/Immunocompromised host
- HIV
- Global health
- Hospital epidemiology/Infection prevention
- Antibiotic stewardship
- Critical care
- Medical education
- Addiction
- Other (please specify)
- Not part of a “track”

What is your gender identify? (select one)

- Female
- Male
- Non-binary
- Other/prefer not to state

Do you identify as transgender?

- Yes
- No
- Other/prefer not to state

What is your race? (select all that apply)

- White
- Black or African American
- American Indian or Alaska native
- Asian
- Hawaiian or Pacific Islander
- Other/prefer not to state

What is your ethnicity? (select one)

- Hispanic
- Not Hispanic
- Other/prefer not to state

—

Clinical experience:

In an average month, how many patients do you see in your practice (including inpatients and outpatients) who use drugs? (select one)

- None that I know of
- 1 to 5 patients
- 6 to 10 patients
- Greater than 10 patients

For patients in your care who use drugs (including inpatients and outpatients), what percentage of the time do you perform each of the following actions? (select percentage for each: 0-24%, 25-49%, 50-74%, 75-100%)

1. Screen for blood-borne viral infections (e.g. HIV, hepatitis B, C)
2. Vaccinate against infections associated with drug use (e.g. hepatitis A, B)
3. Recommend HIV pre- or post-exposure prophylaxis
4. Counsel on strategies to reduce infection risk during drug use
5. Recommend medications to treat substance use disorders (e.g. methadone, buprenorphine, naltrexone, bupropion)
6. Recommend naloxone to treat opioid overdose
7. Refer to inpatient addiction services/resources (e.g. social work, addiction medicine)
8. Refer to outpatient addiction services/resources (e.g. syringe service program, supervised consumption site)

How comfortable are you with each of the following skills? (select one for each: not at all comfortable, slightly comfortable, moderately comfortable, quite comfortable, extremely comfortable)

- 1. Taking a drug use history
  2. Counseling on strategies to reduce infection risk during drug use
  3. Recommending medications to treat substance use disorders
  4. Managing outpatient parenteral antibiotics for people who use drugs

In your opinion, to what extent does each of the following fall within the scope of ID practice?

(select one for each: not at all within scope, slightly within scope, moderately within scope, quite within scope, extremely within scope)

- 1. Taking a drug use history
  2. Counseling on strategies to reduce infection risk during drug use
  3. Recommending medications to treat substance use disorders
  4. Managing outpatient parenteral antibiotics for people who use drugs

Are the following institutional or community resources available to your patients?

(select one for each: available, not available, unsure)

- Inpatients:
  - Social work consultation
  - Addiction medicine consultation
  - Multidisciplinary working group (e.g. endocarditis team)
  - Bridge clinic for linkage to outpatient care
- Outpatients:
  - Addiction medicine clinic
  - Primary care clinic where medications for substance use disorders are prescribed
  - Methadone clinic
  - Syringe service program
  - Supervised consumption site

—

Educational experience:

Prior to ID fellowship, what type of training did you receive in caring for people who use drugs? (select all that apply)

- Medical school-based teaching in addiction medicine
- Residency-based teaching in addiction medicine
- Certification or fellowship in addiction medicine
- Buprenorphine waiver training
- Other (please specify)
- None

During ID fellowship, what type of training have you received (or do you anticipate receiving) in caring for people who use drugs? (select all that apply)

- Informal teaching during patient care encounters
- Fellowship-based lectures/didactics
- Independent reading or coursework (outside of the fellowship)
- Buprenorphine waiver training
- Outpatient parenteral antibiotic training (inclusive of people who use drugs)
- Other (please specify)
- None

Based on your patient care experiences, how helpful would it be to receive education in the following areas? (Select one for each: not at all helpful, slightly helpful, moderately helpful, quite helpful, extremely helpful)

1. Patient-centered communication around drug use
2. Strategies to reduce infection risk during drug use
3. Medication assisted treatment of substance use disorders
4. Overdose prevention and management
5. Institutional/community resources for people who use drugs
6. Outpatient parenteral antibiotic therapy for infections related to drug use

Is there an ID faculty member in your program whom you see as an advocate for people who use drugs? (Yes/No)

Please share any other thoughts or suggestions about ID fellowship education in caring for people who use drugs (free text):

Would you be interested in participating in future focus groups on this topic? (indicate yes/no – if yes, please provide preferred email address)
