# Supplementary material for: Infectious Diseases Fellowship Training in Caring for People Who Use Drugs: A National Assessment of an Emerging Training Need
Source: Open Forum Infect Dis. 2024 Sep 24;11(10):ofae544. doi: 10.1093/ofid/ofae544 (PMC11474980; doi:10.1093/ofid/ofae544)
Supplement: ofae544_Supplementary_Data [file ofae544_supplementary_data.zip › Supplement 2. Focus group interview guide.docx]

Supplement 2. Focus group interview guide

This is a focus group for Infectious Diseases (ID) fellows regarding training and practice experiences in caring for people who use drugs. We encourage everyone over the next hour to unmute and speak freely as if sitting in a room together; the chat will not be monitored. We will begin by reiterating a few items that were shared via email before this session:

1. This meeting will be recorded and transcribed for study purposes
2. Any mention of individual names or institutions will be omitted from the transcript
3. We will take it that in logging in for this session, you have consented to participate
4. Participation remains fully voluntary for the duration of the session and you may log off at any time
5. You may pass on any questions that you prefer not to answer, or contact us after the session if you wish to redact any portions
6. We will randomly award one $25 Amazon gift card at the end of this session as a show of thanks

Are there any questions before we begin? (pause)

We will now proceed with some open-ended questions.

- As an ID consultant, how do you approach the care of a person who uses drugs, as compared with a person who does not use drugs?
  - What do you see encompassed in your role?
  - Do you see your role change when caring for a patient who has, for example, endocarditis related to injection drug use versus endocarditis without injection drug use?
- As an ID fellow, what types of behaviors and attitudes have you seen modeled by faculty when caring for people who use drugs?
  - Have you worked with faculty whom you consider to be advocates for people who use drugs? What traits or actions made those faculty stand out as advocates?
  - Have you observed clinical practices that you plan to emulate?
  - Have you observed clinical practices that don’t resonate with you, or that you plan to approach differently?
- We found in our survey that fewer than half of ID fellows routinely discuss harm reduction, medications for opioid use disorder, naloxone, or HIV pre-exposure prophylaxis in their clinical encounters with people who use drugs. How does this resonate with your experience in fellowship?
  - Does this come as a surprise?
  - Do you see these actions as part of the role of ID providers?
  - Have you observed ID physicians taking on roles that you would consider to be traditionally in the sphere of general or addiction medicine? What are your thoughts on this?
- How does caring for people who use drugs impact your experience of fellowship from the standpoint of burnout, work satisfaction, or sense of purpose?
  - How might ID training programs empower their fellows in caring for people who use drugs?
  - What types of education or support would be beneficial to you in caring for people who use drugs?
- Are there any other thoughts you would like to share on this topic?
