# Supplementary material for: Infectious Diseases Fellowship Training in Caring for People Who Use Drugs: A National Assessment of an Emerging Training Need
Source: Open Forum Infect Dis. 2024 Sep 24;11(10):ofae544. doi: 10.1093/ofid/ofae544 (PMC11474980; doi:10.1093/ofid/ofae544)
Supplement: ofae544_Supplementary_Data [file ofae544_supplementary_data.zip › Supplement 3. Codebook.docx]

Supplement 3. Codebook for focus group transcripts

1. Care of PWUD as a source of burnout - Experiences with patients or systems that cause negative feelings and/or lack of motivation to continue
2. Care of PWUD as a source of morale – Experiences with patients or systems that give a sense of meaning or provide motivation to continue
3. Delineating scope of practice - Negotiating ID’s role and scope of care, as distinct from other fields including addiction medicine, general medicine, etc.
4. Differential care - Situations in which PWUD are treated differently than other patients (whether in a positive or negative manner) by the medical system
5. Educational needs during ID fellowship - Any resources or training that would be beneficial during fellowship in caring for PWUD
6. Expanded ID consultant role - Actions or roles that ID consultants may uniquely take on for PWUD
7. Interdisciplinary care - Experiences in collaborating with colleagues including social work, case management, Addiction Medicine, etc.
8. Lack of institutional/professional support - Constraints in compensation, recognition, or other forms of support in caring for PWUD
9. Social/systemic barriers to care - Situations in which PWUD face barriers in accessing optimal medical care
10. Faculty experiences - Positive and negative experiences in caring for PWUD with faculty, including reflections on teaching and role-modeling
11. Pre-fellowship training - Clinical exposure or training in care of PWUD during medical school or residency

Abbreviations: Infectious Diseases (ID), people who use drugs (PWUD), medications for opioid use disorder (MOUD)
